# The prevalence of depression, anxiety, and sleep disturbances among medical students and resident physicians in Iran: A systematic review and meta-analysis

**DOI:** 10.1371/journal.pone.0307117

**Published:** 2024-08-23

**Authors:** Arman Shafiee, Mohammad Mobin Teymouri Athar, Niloofar Seighali, Mohammad Javad Amini, Hamed Hajishah, Razman Arabazadeh Bahri, Amirhossein Akhoundi, Maryam Beiky, Nastaran Sarvipour, Saba Maleki, Atefeh Zandifar, Mahmood Bakhtiyari

**Affiliations:** 1 Non-Communicable Diseases Research Center, Alborz University of Medical Sciences, Karaj, Iran; 2 Student Research Committee, School of Medicine, Alborz University of Medical Sciences, Karaj, Iran; 3 Student Research Committee, School of Medicine, Shahid Beheshti University of Medical Sciences, Tehran, Iran; 4 Student Research Committee, School of Medicine, Islamic Azad University of Medical Sciences, Tehran, Iran; 5 School of Medicine, Tehran University of Medical Sciences, Tehran, Iran; 6 Faculty of Medicine, Kerman University of Medical Sciences, Kerman, Iran; 7 School of Medicine, Guilan University of Medical Sciences (GUMS), Rasht, Guilan Province, Iran; 8 Department of Psychiatry, Alborz University of Medical Sciences, Karaj, Iran; Shahrood University of Medical Sciences, ISLAMIC REPUBLIC OF IRAN

## Abstract

**Background:**

We sought to conduct this comprehensive systematic review and meta-analysis to assess the prevalence of depression, anxiety, and sleep disturbance in Iranian medical students and resident physicians.

**Methods:**

A systematic search was conducted on 23 December 2023 in PubMed/MEDLINE, Web of Science, Scopus, and Iranian national databases. We pooled the prevalence of individual studies using the random effect model.

**Results:**

Our systematic search showed 36 articles that meet the eligibility criteria. Most included studies were cross-sectional. The most used questionnaire to assess depression, anxiety, and sleep disturbance were Beck Depression Inventory (BDI), The Depression, Anxiety and Stress Scale—21 Items (DASS-21), and The Pittsburgh Sleep Quality Index (PSQI), respectively. The overall prevalence of depression, anxiety, and sleep disturbance among Iranian medical students were 43% (95%CI: 33%–53%%, I2 = 98%), 44% (95%CI: 31%–58%%, I2 = 99%), 48% (95%CI: 39%–56%%, I2 = 97%), respectively. The results of subgroup and meta-regression analyses showed questionnaires used and the place of the medical school were significantly associated with the prevalence of aforementioned outcomes. Funnel plot and Begg’s regression test did not show a significant source of funnel plot asymmetry for depression, anxiety, and sleep disturbance.

**Conclusion:**

In conclusion, our study showed that nearly half of the medical students had some type of depression, anxiety, and sleep disturbance problems. To address this serious national public health issue, efficient preventive measures, routine screenings, and prompt interventions are required.

## Introduction

Mental health is defined as the "state of well-being with realizing own abilities, coping with the normal stresses of life, working productively, and being able to contribute to the community". Mental disorders are one of the important public health concerns and their prevalence increased in the last decade. The most common mental disorders are anxiety and depression [1]. The global prevalence of depression and anxiety disorders was reported at 3.4% and 3.8% in the last update of 2021, respectively [2]. They were also found to be related to sleep problems [3–5]. Poor sleep quality is a leading factor to increases the risk of secondary mental illness, including anxiety and depression [6, 7] and decreases the efficiency of rest, an individual’s daily ability, and quality of life [8, 9].

As medical students often face stressful situations, they are more likely to suffer from anxiety [10], depression [9, 11], and poor sleep quality which may affect their academic performance [12, 13]. The global prevalence of depression was reported in a recent meta-analysis threefold higher in medical students compared to their peers of similar age [14].

Previous studies in different parts of Iran indicated various prevalences of anxiety, 42.5% at Qazvin University of Medical Sciences [15], 29.61% at Tehran University of medical sciences [16], 100% at Shahid-Beheshti University of medical sciences [17]. Prevalence of depression was reported at 49.6% at Qazvin University of medical sciences [15], 26.1% at Tehran University of medical sciences [16]; and poor sleep quality was 78.8% at Babol University of medical sciences [18], 40.6% of Zanjan University of Medical Sciences [19], and 57.2% at Shiraz University of medical sciences [20]. Besides, inconsistent data for different majors and degrees of education were reported in studies too [19, 21, 22].

Although several studies have been conducted among Iranian medical students regarding the assessment of poor sleep quality, anxiety, and depression prevalence [16, 19, 22], no national systematic review or meta-analysis has yet been conducted to examine the prevalence of mental disorders and poor sleep quality in Iranian medical students. Regular investigations and screening for sleep and mental disorders may help control the risk of developing psychological and physical symptoms in Iranian medical students. Thus, due to inconsistent data and the lack of evidence from a national meta-analysis study, we set out to conduct this comprehensive systematic review and meta-analysis to assess the prevalence of depression, anxiety, sleep disturbance, and metal disorder in Iranian medical students and resident physicians.

## Methods

We carried out a systematic review following the Cochrane Manual for Systematic Reviews of Interventions’ recommendations and the Preferred Reporting Items for Systematic Reviews and Meta-Analyses (PRISMA) criteria [23, 24].

### Search strategy

We conducted a systematic search in Medline (via PubMed), Web of Science, Scopus, MagIran, Elmnet, and IranDoc from their commencement until 23 December 2023. Furthermore, we screened the references of eligible articles to include possible missed articles. The optimal strategy for our thorough search was developed by combining the following keywords with the Boolean operators: depression, depressive syndrome, anxiety, sleep disorder, sleep disturbance, medical students, and resident physicians. In the Supplemental material, a detailed explanation of the search procedure for each database has been provided.

### Eligibility criteria

Using the PICOT acronymous, the inclusion criteria were: 1) Population: Iranian medical students and resident physicians; 2) Intervention: not applicable; 3) Comparison: not applicable; 4) Outcome: the prevalence of depression, anxiety, and sleep disturbance.; 5) Type of Study: Observational studies which mostly included cross-sectional studies. Conference abstracts and theses were also included since this study was aimed to provide a nationwide systematic review. Only studies that defined a cut-off value for reporting prevalence were included. Review studies, opinion studies, and letters to the editor devoid of any pertinent information all met the exclusion criteria.

### Screening and data extraction

The full texts of the papers were checked after the title and abstract were initially evaluated. Disagreements were resolved through discussions. A spreadsheet in Excel was used to extract the data. The following data were extracted: Author, Year, Country, Type of publication, Type of study, Medical school, Population, Total number of participants, Age, Male (%), Questionnaire, Rate of response, and Cutoff values.

### Questionnaires

The following questionnaires were used for assessing the prevalence of depression among Iranian medical students: The Depression, Anxiety and Stress Scale—21 Items (DASS-21), Beck Depression Inventory (BDI), The Minnesota Multiphasic Personality Inventory (MMPI), University student depression inventory (USDI), General Health Questionnaire – 28 (GHQ-28), Symptom Checklist 90-R (SCL-90-R), and self-designed questionnaires defined as others.

Included articles assessed anxiety using the following questionnaires: The Depression, Anxiety and Stress Scale—21 Items (DASS-21), Beck Anxiety Inventory (BAI), The Minnesota Multiphasic Personality Inventory (MMPI), State-Trait Anxiety Inventory (STAI), Zung Self-Rating Anxiety Scale (SAS), and self-designed questionnaires defined as others. Only two questionnaires were used to assess sleep disturbance: The Pittsburgh Sleep Quality Index (PSQI) and the 7-item Insomnia Severity Index.

### Quality assessment

We used the modified version of the Newcastle-Ottawa Scale (NOS) to assess the quality of cross-sectional studies [25]. The scale contains 6 domains (Representativeness, Sample Size, Non-Respondents, Valid Measurement Tool, Valid Statistical Methods, Peer reviewed). Representativeness, sample size, and valid statistical methods were assessed as defined by NOS. Non-respondents were defined as a response rate of at least 80%. Valid measurement was those questionnaires previously validated. Peer reviewed domain was rated up if the study was published in a peer-reviewed journal. The overall rate of 5 and 6 shows a low risk of bias.

### Data synthesis

We used the random effect model because of the different demographics and characteristics between studies to pool the prevalence of depression, anxiety, and sleep disturbances among Iranian medical students and resident physicians. Between studies, heterogeneity was calculated using I2 statistics. I2 values >75% were defined as high heterogeneity. In the case of heterogeneity, several subgroup analyses, meta-regression, and sensitivity analyses were done. We performed subgroup analyses by different assessment questionnaires, stage (Pre-clinical or Clinical courses), medical school, and degree (medical student or resident physician). A meta-regression was done to evaluate the correlation between outcomes and study covariates; considering publication date as covariate of interest. All statistical analyses and graphics were carried out using StataCorp. 2023. Stata Statistical Software: Release 18. College Station, TX: StataCorp LLC.

## Results

### Characteristics

**Fig 1:**
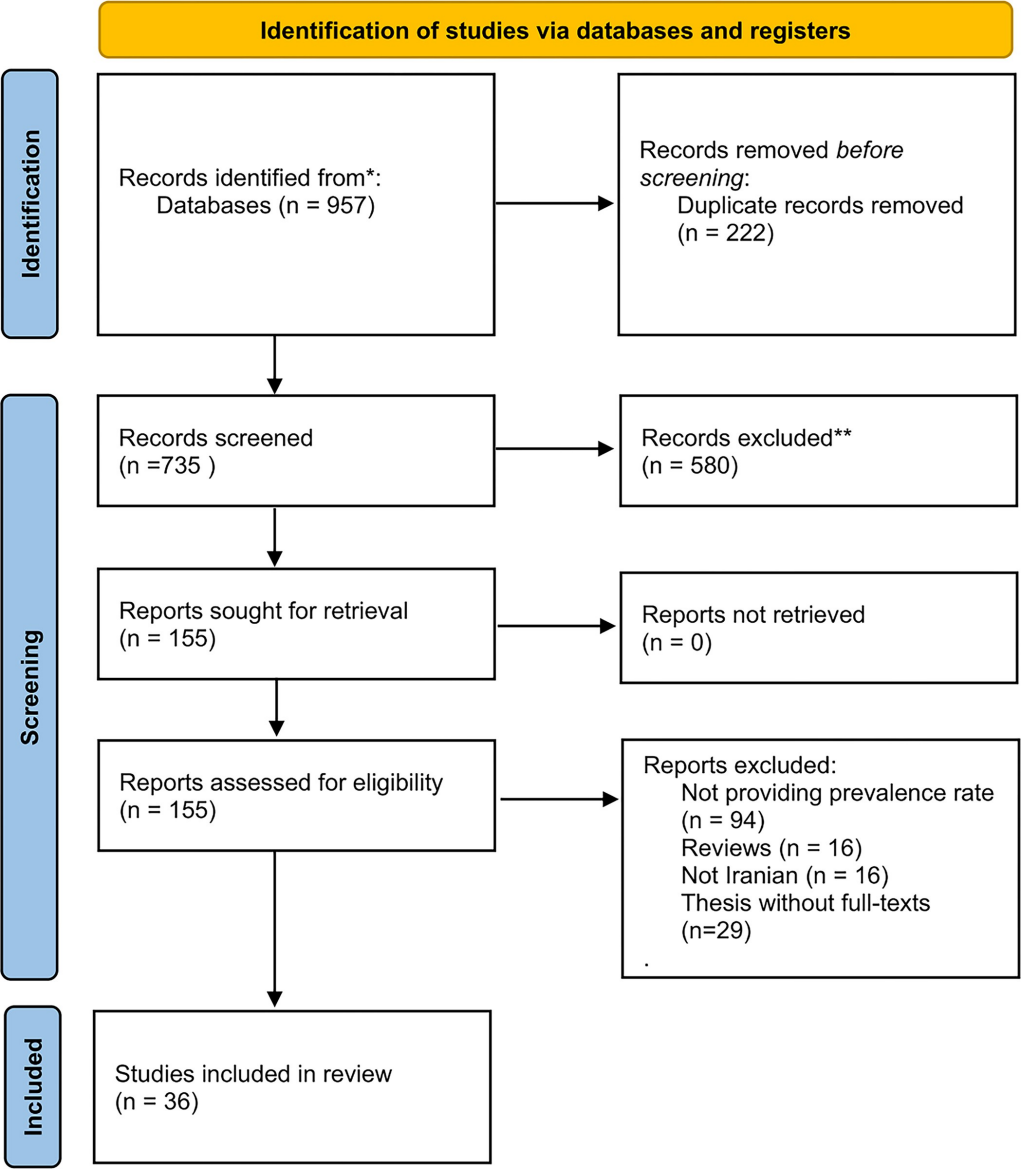
PRISMA flow diagram for article selection.

Our search result identified 1283 articles. Initially, 532 articles were removed after screening based on title and abstract. Finally, 36 studies were included based on our eligibility criteria [15, 16, 18–20, 22, 26–55] (Fig 1). Twenty-five articles were peer-reviewed publications published in international databases; 11 studies were peer-reviewed studies published in Iranian databases. The detailed characteristics of each study are available in Table 1. Most studies were cross-sectional regarding study design.

**Table 1:** Characteristics of the included studies.

Author	Year	Study design	Medical School	Population	Total Patients (Total/ Those who respond)	Age (mean ± SD)	Male (%)	Sampling method	Questionnaire	Rate of response	Assessment scales and cutoff value
Abdali et al. [26]	2019	Cross-sectional	Semnan University of Medical Sciences	Medical Students (combined)	259	NR	NR	systematic random sampling	PSQI	NR	6 or more indicating sleep quality disorders
Aghajani Liasi et al. [27]	2021	Cross-sectional	Tehran Islamic Azad University of Medical Sciences	Medical Students (clinical)	123 / 120 stager	24.4 + 2.06 (range = 20–34)	19.30%	counting	DASS-42	97.50%	Depression: 0–9 normal, 10–13 mild, 14–20 moderate, 21–27 severe, and 28+ extremely severe;
											Anxiety: 0–7 normal, 8–9 mild, 10–14 moderate, 15–19 severe, and 20+ extremely severe; and Stress: 0–14 normal, 15–18 mild, 19–25 moderate, 26–33 severe, and 34+ extremely severe.
Ashouri et al. [85]	2023	Cross-sectional	Tehran University of Medical Sciences	Residents	180/130	30.23 ± 2.47	63.10%	NR	BAI	71.40%	NR
Ghoreishi et al. [19]	2008	Cross-sectional	Zanjan University of Medical Sciences	Medical Students	280/234	23 ± 2.815 range = 18–31	0.388	counting	PSQI	80%	5 for PSQI (over 5 = poor sleep quality)
Haghighi, M. [32]	2019	Cross-sectional	Hamadan University of Medical Sciences	Medical Students	(256/207)	22.04 (2.74)	51%	NR	Beck Depression Inventory (BDI-II); State-Trait Anxiety Inventory (STAI); 7-item Insomnia Severity Index	80.50%	Scores between 19 and 29 indicate moderate depression, scores between 30 and 63 indicate severe depression; Scores on the STAI> 30 indicate moderate anxiety and scores> 45 reflect severe anxiety; 7-item Insomnia Severity Index
Jafari et al. [33]	2017	Cross-sectional	Shiraz University of Medical Sciences	Medical Students	783/477	NR	49.80%	NR	Depression Anxiety Stress Scales-21 (DASS-21)	61%	Normal = (0–4 for depression, 0–3 for anxiety) mild = (5–6 for depression, 4–5 for anxiety) moderate = (7–10 for depression, 6–7 for anxiety) severe = (11–13 for depression, 8–9 for anxiety) extremely severe = (>14 for depression, >10)
Janatmakan Amiri, A. [34]	2020	Cross-sectional	Mashhad University of Medical Sciences	Medical Students	(310/300)	21:94 ± 2:28	165 (55%)	stratified random sampling	Pittsburgh Sleep Quality Index (PSQI) and self-design (DASS-21)	96%	21[higher than 21 = sleep disorders, lower than 21 = sleep disorders]
Javadi et al. [15]	2019	Cross-sectional	Qazvin University of Medical Sciences	Medical Students	253	22.32±2.85	81 (31.9%)	stratified random sampling	BDI, Cattle anxiety questionnaire, self-designed questionnaire for sleep quality assessment	NR	BDI> 16; Cattle anxiety = NA; Sleep disturbance> 0
Khorvash, F. [35]	2013	Cross-sectional	Universities of Medical Sciences of Isfahan, Gilan, Sanandaj and Kashan	Residents	(400/370)	163 subjects (44.1%) were younger than 30, 191 (51.6%) between the age of 30 and 40 and 28 participants were older than 40 years old	42.40%	category sampling method	Zung anxiety scale	92.30%	20-44: Normal range • 45-59: Mild to moderate anxiety levels • 60-74: Marked to severe anxiety levels • 75-80: Extreme anxiety levels.
Maghsoudi, S. [36]	2022	Cross-sectional	Isfahan university of medical sciences	Medical Students	(106/100)	23.2 (±4.8 SD years)	32%	block sampling	The Pittsburgh Sleep Quality Index (PSQI)	94.30%	A total score higher than five for the questionnaire means poor sleep quality
Malek et al. [37]	2019	Cross-sectional	Tehran University of Medical Sciences	Medical Students	(107/96)	21	42.7	convenience sampling	Minnesota Multiphasic Personality Inventory (MMPI-2)	89.70%	NR
Miri et al. [38]	2017	Cross-sectional	94 Beheshti University of Medical Sciences	Medical Students	549	24.2±5.9	36.60%	NR	GHQ-28	NR	23 [higher than 23 = mental disorders, lower than 23 = mental health]
			83 Shiraz University of Medical Sciences								
			Mashhad, Yazd, Gorgan, Rafsanjan, and Kordestan University of Medical Sciences								
Mohammadbeigi, A. [40]	2016	Cross-sectional	Qom University of Medical Sciences	Medical Students	(380/363)	21.8 ±3.2	30.9	stratified random sampling	Pittsburgh sleep quality questionnaire	95.50%	The total score of sleep quality was categorized based on the lower or higher 5 and labeled as normal and poor, respectively
Moudi et al. [18]	2014	Cross-sectional	Babol University of Medical Sciences	Medical Students	153	20–35	50.30%	stratified random sampling	PSQI	NR	Higher than 6 indicated sleep disturbances
Naderi [42]	2023	Prospective descriptive-analytic	Isfahan University of Medical Sciences	Medical Students (combined)	110/106	49.21±9.15	58.5%	stratified random sampling	PSQI	NR	Higher than 6 indicated sleep disturbances
Nakhostin-Ansari, A. [44]	2020	Cross-sectional	Tehran University of Medical Sciences	Medical Students	(500/323)	23.73 (SD = 1.62)	47.70%	NR	Beck Anxiety Inventory (BAI) and Beck Depression Inventory (BDI)	64.60%	BAI = 10 and BDI = 13
Pournik et al. [45]	2020	Cross-sectional	Tehran University of Medical Sciences	Medical Students (basic sciences)	250/154	19.1 (± 0.99)	44.20%	NR	PSQI	62%	Cutoff point of 5 is administered to assign participants either to good or poor sleep quality
Rahmati, F. [47]	2019	Prospective Cross-sectional	Shahid Beheshti University of Medical Sciences	Emergency medicine residents	(99/99)	33.93 ± 5.92	43.40%	census method	MMPI	100	NR
Rezaei et al [16]	2018	Cross-sectional	Tehran University of Medical Sciences	Medical Students (first second third year)	616 / 553	21.69 ± 1.11 range = 19–27	48.50%	NR	Pittsburgh Sleep Quality Index (PSQI) Depression, Anxiety and Stress Scale-21 (DASS21)	89.70%	5 for PSQI (over 5 = poor sleep quality) DASS-21: Depression [normal (0–9), mild (10–13), moderate (14–20), severe (21–27), and extremely severe (28+)] Anxiety [normal (0–7), mild (8–9), moderate (10–14), severe (15–19), and extremely severe (20+)] Stress [normal (0–14), mild (15–18), moderate (19–25), severe (26–33), and extremely severe (34+)]
Sadr, S. S. [48]	2014	Cross-sectional	Shahid Beheshti University of Medical Sciences	Medical Students in the Internship Stage	(100/100)	NR	0%	available sampling	Beck Depression Inventory	NR	Higher than 9
Sahraian [20]	2010	Cross-sectional	Shiraz University of Medical Sciences	Medical Students (combined)	159	21.5(2.67)	49.70%	NR	PSQI	NR	NR
Shadzi et al. [22]	2020	Cross-sectional	Shiraz University of Medical Sciences	Medical Students (combined)	487 / 402	22.4 (±2.18)	0.497	stratified random sampling	21-item Depression Anxiety Stress Scale (DASS-21) Pittsburgh Sleep Quality Index (PSQI)	82.5%	DASS-21: depression (normal: scores 0–9, moderate: scores 10–20, and severe: scores 21–42), anxiety (normal: scores 0–7, moderate: scores 8–14, and severe: scores 15–42), stress (normal: scores 0–14, moderate: scores 15–25, and severe: scores 26–42) PSQI: 6 (over 6 = poor sleep quality)
Shariatpanaahi, M. V. [51]	2007	Cross-sectional	Islamic Azad University of Tehran	Medical Students (clinical)	(192/192)	24.571.6	0	NR	BDI	NR	A score of 10 or higher was considered as depression
Teimouri et al. [53]	2021	Cross-sectional	Isfahan University of Medical Sciences	Medical Students (combined)	NR/ 290	NR	116 (40%)	block sampling	Pittsburgh Sleep Quality Index (PSQI)	aproximately 80% (less)	5 [higher than 5 = poor sleep quality]
Yazdi, Z. [55]	2016	Cross-sectional	Qazvin University of Medical Sciences	Medical Students (combined)	(325/285)	22.8 ±1.74	47.4	NR	PSQI	87.7	more than 5

### Depression

**Fig 2:**
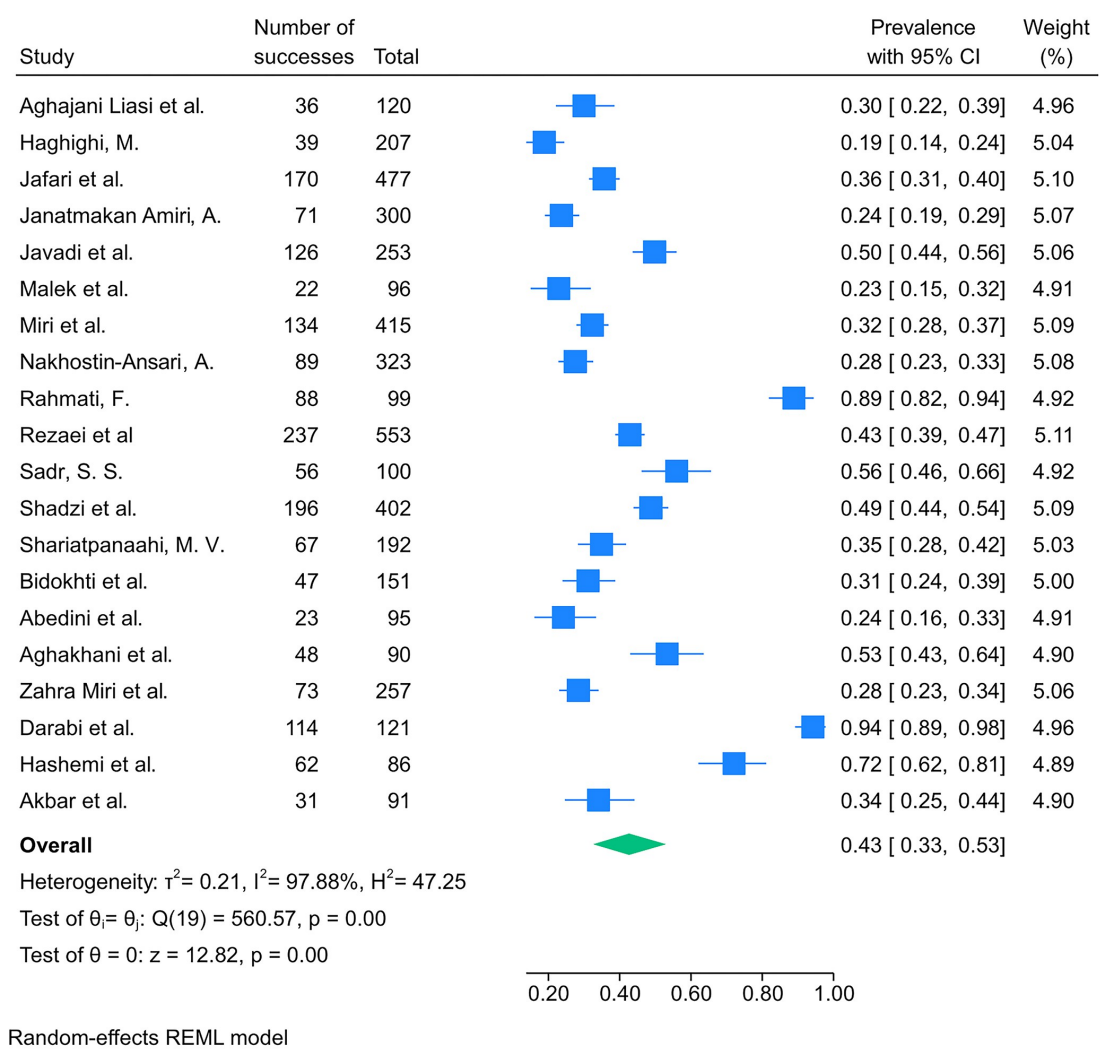
The results of the meta-analysis showing the prevalence of depression among Iranian medical students and resident physicians.

From the included studies, 20 reported data regarding the prevalence of depression among Iranian medical students and resident physicians. Studies were published between 2002 and 2021. One study reported the prevalence of depression among resident physicians. The most questionnaire used among the included studies was BDI with 9 articles. Three studies specifically investigated the aforementioned outcome among pre-clinical medical students. The results of our meta-analysis showed a pooled prevalence of depression was 43% (95%CI: 33%–53%%, I2 = 98%) among Iranian medical students and resident physicians (Fig 2). Furthermore, the pooled prevalence of moderate to severe depression was 25% (95%CI: 14%–38%%, I2 = 98%). The result of our subgroup analysis revealed the questionnaire used for assessing depression and the place of medical school were significantly associated with the prevalence of depression among medical students (S1 and S2 Figs). Furthermore, the meta-regression analysis showed that the prevalence of depression was not significantly associated with the year of publication, showing an independent positive correlation (p-val = 0.89).

### Anxiety

**Fig 3:**
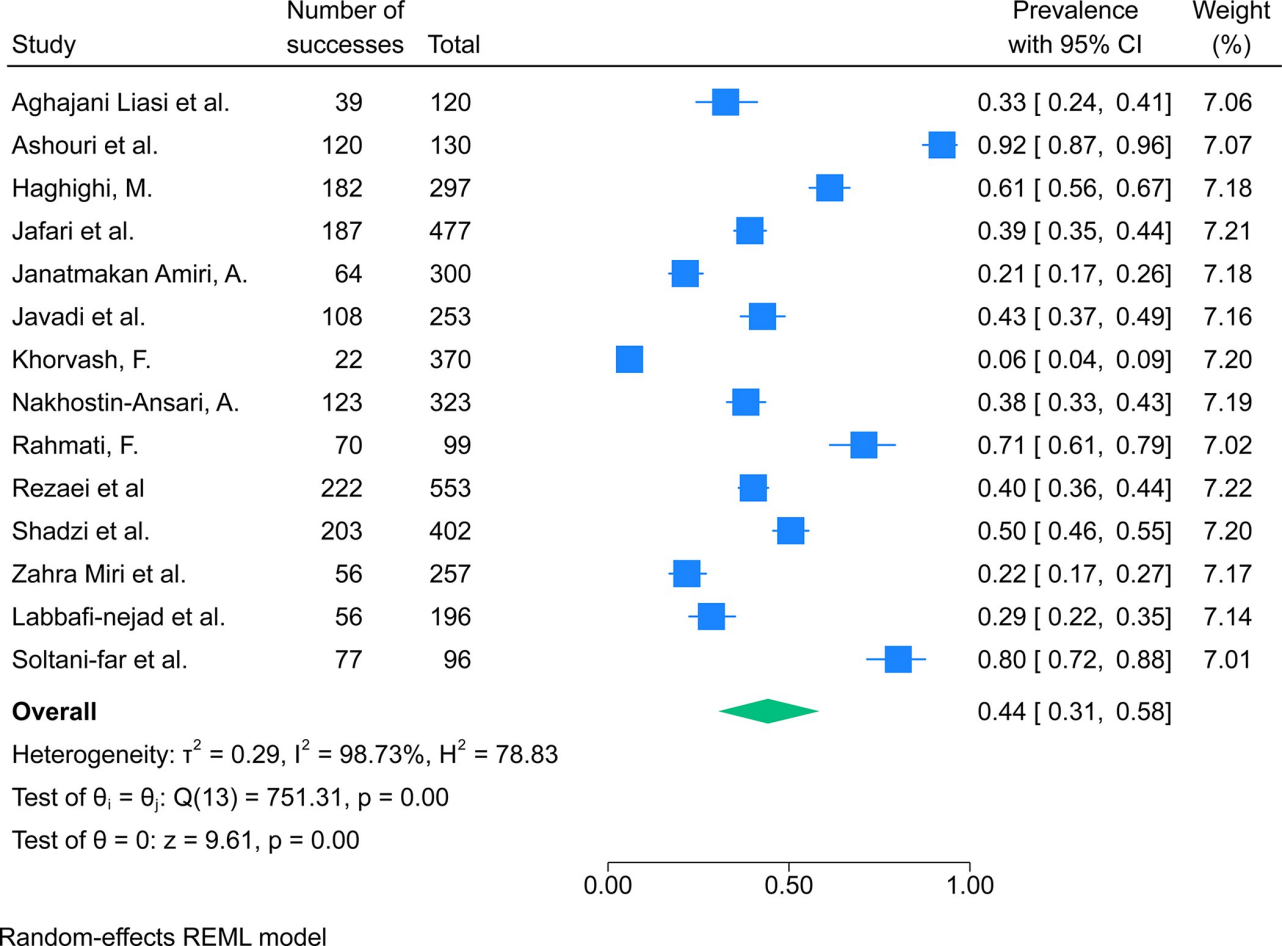
The results of the meta-analysis showing the prevalence of anxiety among Iranian medical students and resident physicians.

From the included studies, 14 reported data regarding the prevalence of anxiety among Iranian medical students and resident physicians. Studies were published between 2010 and 2023. Three studies reported the prevalence of anxiety among resident physicians. The most questionnaire used among the included studies was DASS with 10 articles. Two studies specifically investigated the aforementioned outcome among pre-clinical medical students. The results of our meta-analysis showed a pooled prevalence of anxiety was 44% (95%CI: 31%–58%%, I2 = 99%) among Iranian medical students and resident physicians (Fig 3). Furthermore, the pooled prevalence of moderate to severe anxiety was 28% (95%CI: 15%–43%%, I2 = 98%). The result of our subgroup analysis revealed the questionnaire used for assessing anxiety and the place of medical school were significantly associated with the prevalence of anxiety among medical students (S3 and S4 Figs). Furthermore, the meta-regression analysis showed that the prevalence of anxiety was not significantly associated with the year of publication (p-val = 0.51).

### Sleep disturbance

**Fig 4:**
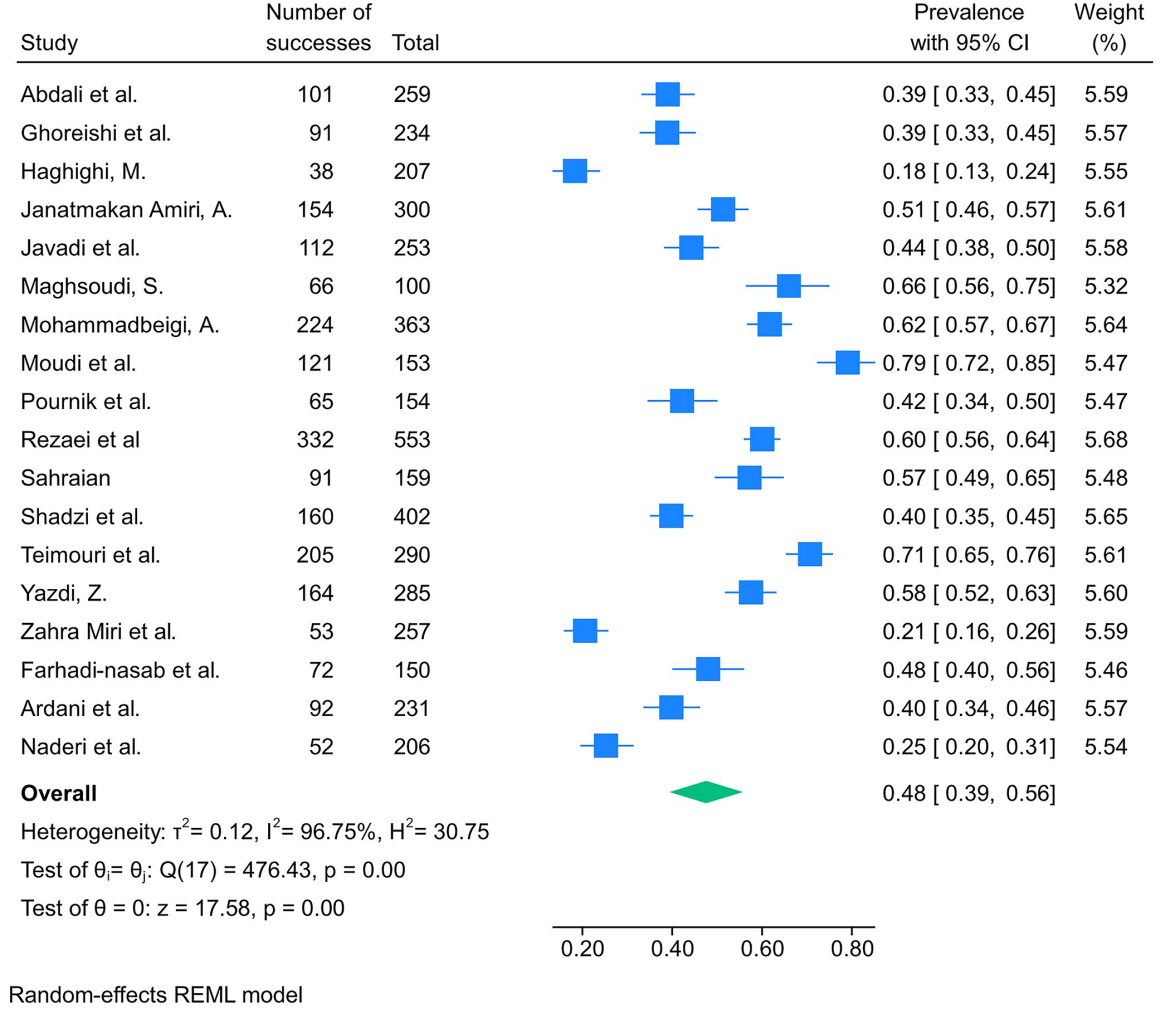
The results of the meta-analysis showing the prevalence of sleep disturbance among Iranian medical students.

From the included studies, 19 reported data regarding the prevalence of sleep disturbance among Iranian medical students and resident physicians. Studies were published between 2007 and 2023. There was no study reporting the prevalence of sleep disturbance among resident physicians. The most questionnaire used among the included studies was PSQI. Two studies specifically investigated the aforementioned outcome among pre-clinical medical students. The results of our meta-analysis showed a pooled prevalence of sleep disturbance was 48% (95%CI: 39%–56%%, I2 = 97%) among Iranian medical students (Fig 4). The result of our subgroup analysis revealed the questionnaire used for assessing sleep disturbance, the year of study, and the place of medical school were significantly associated with the prevalence of sleep disturbance among medical students (S5 Fig). Furthermore, the meta-regression analysis was not associated with the year of publication (p-val = 0.50).

### Quality assessment and publication bias

The quality assessment scores ranged between 4 to 6. Most studies showed a low risk of bias. However, there were 7 studies with moderate quality. Detailed results of each study’s quality assessment are available in Supporting material.

**Fig 5:**
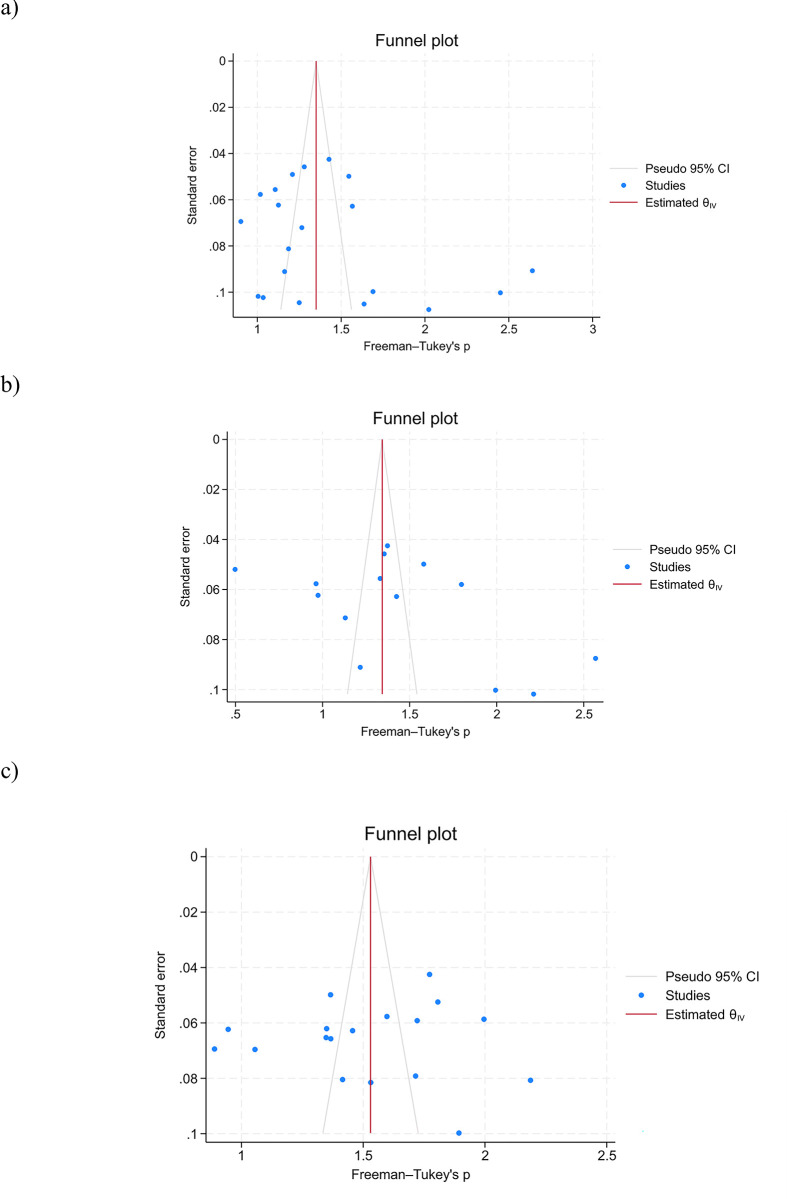
a) Funnel plot for the prevalence of depression; b) Funnel plot for the prevalence of anxiety; and c) Funnel plot for the prevalence of sleep disorder.

The funnel plot and Begg’s regression test did not show a significant source of funnel plot asymmetry for depression (p-value = 0.31), anxiety (p-value = 0.23), and sleep disturbance (p-value = 0.94) (Fig 5).

## Discussion

According to the World Health Organization (WHO), mental health problems are on the rise. The prevalence of mental health issues is increasing day by day with anxiety disorders and depression being the most common globally affecting 375 and 246 million individuals worldwide, respectively. Moreover, the COVID-19 pandemic has caused a significant increase in the prevalence of these disorders [56]. These issues not only impact the individuals and their social environment but also have far-reaching consequences for society as a whole, mostly due to economic costs [57]. Therefore, the diagnosis and providing care for those affected by these situations is of utmost importance.

Different segments of society may experience the impact of these disorders to varying degrees, depending on their lifestyle and level of exposure to relevant risk factors. Given their significance as the foundation of future healthcare systems, medical students’ mental health is currently a topic of intense global attention. Medical students must maintain appropriate psychological well-being, not only for their own sake but also for the benefit of their future patients [58]. Recent research conducted globally has revealed alarmingly high rates of mental distress among medical students, prompting policymakers to take proper action [59].

Medical education is one of the most demanding academic programs. As a result, students pursuing this type of education are at increased risk of experiencing negative mental health outcomes. This is partly due to the rigorous selection process of medical schools to identify intelligent and devoted individuals. Significant time and emotional commitment along with stress can impose a detrimental effect on their mental health [27]. Previous research in Western countries has reported that in comparison with the general population, those in the field of medicine show a higher frequency of mental morbidity [60, 61].

It has been documented through numerous studies that initially, medical students are comparable to their non-medical peers in terms of mental health, however, they tend to experience a decline in their mental health throughout their education [61, 62].

The reason for this vulnerability is probably multifaceted and involves a combination of environmental and individual factors. Medical education is a lengthy process where students must face multiple stressors. Previous studies have classified the stressors into three categories: academic stressors, social stressors, and health-related stressors.

Academic or educational stressors are those directly related to the extensive educational years which demand a lot of time and effort [63]. These factors include performance in the exams, demanding educational curriculum with an abundance of information, difficulty accessing the proper material for studying, competition with their peers, an overwhelming workload, not having adequate time for recreational activities, and expectations both from themselves and others, responsibilities in clinical rotations including presence in hospital wards, witnessing the death and suffering of the patients.

On the other hand, a tough and time-consuming academic life deprives students of opportunities for self-improvement and enhancing their social life. Psychosocial stressors including loneliness, homesickness, social circumstances of the country, financial problems, adjustment to the university and hospital environment, and boredom are additional factors. Moreover, some influencing factors are related to the health condition of the students including disturbance in the sleep pattern along with lack of physical exercise, and smoking are among those [64, 65].

The process of becoming a medical doctor in Iran starts with “Konkour”, a difficult and stressful university entrance exam with hundreds of thousands of participants graduating from high school held to identify the most intelligent and hard-working students for admission to higher education [66]. Iranian medical education curriculum consists of four major stages: basic sciences, pathophysiology, clerkship, and the clinical internship which take about 7 years to complete. Moreover, entering each phase is only possible after passing comprehensive exams which encompass a large amount of study material, causing anxiety and stress [67]. The variations in the prevalence of anxiety among medical students in Iran from different universities can be influenced by several factors. Firstly, the city in which the university is located may play a role due to differences in environmental stressors, access to mental health resources, and overall lifestyle factors. Secondly, the variety of assessment tools used to measure anxiety can lead to differences in reported prevalence rates, as different tools may capture different aspects of anxiety [44]. Thirdly, differences in the curriculum and teaching methods among universities can impact students’ stress levels and psychological well-being [68]. Finally, the disparity in the level of equipment and facilities available at different universities may affect students’ overall experience and well-being, potentially contributing to variations in anxiety prevalence. These factors collectively contribute to the observed differences in anxiety prevalence among medical students in Iran and highlight the need for comprehensive research to address and understand these variations. The national university entrance exam in Iran, known as Konkour, differs from those in other countries in several ways. Unlike some other countries where the entrance examination is centralized but admission is decentralized, Iran has a centralized examination and admission system [69]. Additionally, the Konkour exam is a comprehensive 4.5-hour multiple-choice test that covers all subjects taught in Iranian high schools, and its results solely determine admission to medical school and other fields. This differs from the entrance examinations in some other countries, where high school records and other factors may also be considered in the admission process. Furthermore, the competition for university places in Iran is intense, as the exam is highly competitive and the number of public university spots is limited. These differences highlight the unique nature of the Konkour exam and its significant impact on the academic destinies of Iranian students compared to entrance examinations in other countries [70].

The negative consequences of mental health issues such as stress, depression, and anxiety disorders can significantly impact a student’s overall well-being and affect their ability to learn and perform academically. Compromised concentration and decision-making skills play a major role in impairing the individual’s learning process. Moreover, making a rational relationship with patients is more difficult since these conditions lower self-confidence. In the U.S., studies have demonstrated high degrees of cynicism and indifference toward patients in medical students [61, 71]. Medical errors are of great concern as they are likely with diminished focus and competency. Furthermore, studies have shown a higher tendency toward substance abuse among medical students compared with control groups [72, 73].

In Iran, Social stigma and inadequate follow-up care are possible reasons for the higher prevalence of psychiatric disorders. Many students avoid seeking help through formal and professional psychiatric consultations [74].

There is a bidirectional relationship between psychiatric illnesses and sleep disorders, particularly depression and anxiety disorders. [75]. Sleep serves as a vital physiologic component of life and its disruption causes detrimental physical and mental health effects.

Medical students might be prone to sleep disturbances as a result of the demanding nature of their studies which requires long hours of study and clinical responsibilities such as night shifts. This situation impedes proper training, endangering future patients’ safety [76, 77].

Studies have shown that insufficient sleep and sleep pattern disturbances influence an individual’s capacity to learn and function properly in situations that require concentration, attention, and memory [78, 79]. In addition, medical students suffering from poor sleep are at a greater risk of burnout.

As of the writing of this article, the world is still facing the predicaments caused by the COVID-19 pandemic. The virus had a profound impact on various crucial areas such as economics, politics, and society, causing many individuals worldwide to struggle with mental health issues. Students, in particular, have been affected by this psychological toll to a great extent. Closure of universities, restriction of social interactions and outdoor activities, lockdown for prevention of the spread of the disease, and sudden alterations in the normal and daily lifestyle are among the contributing factors. Medical students have been disproportionately affected by this unprecedented situation and prevalence of COVID-19, particularly in countries such as Iran since they were involved in the provision of health care on the frontline of the pandemic [80]. Studies performed around the globe have demonstrated the incidence of mental health disorders among medical students during the pandemic [44, 81].

These findings have several possible important implications. Establishing systems for monitoring the mental health of medical students and residents could prevent further complications through early diagnosis and appropriate intervention [82].

Our research has strengths in several aspects. To the best of our knowledge, this is the most comprehensive and updated assessment of the prevalence of mental health disorders among Iranian medical students and resident physicians. Our search protocol was precisely designed encompassing several databases and the result of our comprehensive database search included a considerable number of studies including research articles and theses both in English and Farsi. Moreover, we introduced three components of mental health including depression, anxiety, and sleep disturbances. Furthermore, the prevalence of mental disorders among resident physicians was evaluated in addition to medical students.

Despite the strengths, the results of our study should be interpreted considering some limitations. Some of the studies included medical sciences students in various majors such as dentistry and nursing and not only the students of medicine as a separate group. Some included studies were published in Iranian journals. Therefore, compared with international journals, differences in the process of peer review potentially cause bias in terms of the quality of evidence. Moreover, some of the studies have not reported the exact number of participants with mental disorders, only providing the mean and standard deviation of the scores obtained from questionnaires.

In conclusion, our study revealed that nearly half of the medical students experienced depression, anxiety, and sleep disturbances. To address this significant public health issue, it is crucial to implement efficient preventive measures, conduct routine screenings, and provide prompt interventions. Since a specific and well-designed study is missing on the topic of Iranian medical students and resident physicians mental health [83, 84], we suggest specific steps for future research, including longitudinal cohorts to understand long-term effects, developing targeted interventions for Iranian medical students, providing policy recommendations for integrating mental health support within the medical education system, and ensuring cultural sensitivity in mental health programs [84].

## Supporting information

S1 Table PRISMA 2020 checklist. (DOCX)

S2 Table Search strategies for online databases. (DOCX)

S3 Table The characteristics of included studies from Iranian national databases. (DOCX)

S4 Table Quality assessment for each included study. (DOCX)

S1 Fig Results of subgroup analysis for depression. (PDF)

S2 Fig Results of meta-analysis analysis for moderate to severe depression. (PDF)

S3 Fig Results of subgroup analysis for anxiety. (PDF)

S4 Fig Results of meta-analysis analysis for moderate to severe anxiety. (PDF)

S5 Fig Results of subgroup analysis for sleep disorder. (PDF)
