# Supplementary material for: The prevalence of depression, anxiety, and sleep disturbances among medical students and resident physicians in Iran: A systematic review and meta-analysis
Source: PLoS One. 2024 Aug 23;19(8):e0307117. doi: 10.1371/journal.pone.0307117 (PMC11343466; doi:10.1371/journal.pone.0307117)
Supplement: S2 Table — (DOCX) [file pone.0307117.s002.docx]

**Supporting Table 2.** Search strategies for online databases

| **MEDLINE (via PubMed)** | 1- “Depressive Disorder”[mh] OR “Depression”[mh] OR “Anxiety”[mh] OR “Sleep Wake Disorders”[mh] OR “Mental Disorders “[mh] OR “Depression “[tiab] OR “Depressive “[tiab] OR “Depress*”[tiab] OR “Anxiety”[tiab] OR “Anxieties “[tiab] OR “Anxiousness”[tiab] OR “Nervousness”[tiab] OR “Hypervigilance”[tiab] OR “Sleep”[tiab] OR “Mental Disorders”[tiab] OR “Mental Health”[tiab] OR “insomnia”[tiab]  2- “Medical students”[tiab] OR “Medical student”[tiab] OR “resident doctors”[tiab] OR “resident doctor”[tiab] OR “resident physicians”[tiab] OR “resident physician”[tiab] OR “Residency “[tiab]  3- Iran |
| --- | --- |
| **Scopus** | 1- “Depression “OR “Depressive “OR “Depress*”OR “Anxiety”OR “Anxieties “OR “Anxiousness”OR “Nervousness”OR “Hypervigilance”OR “Sleep”OR “Mental Disorders”OR “Mental Health”OR “insomnia”  2- “Medical students”OR “Medical student”OR “resident doctors”OR “resident doctor”OR “resident physicians”OR “resident physician”OR “Residency”  3- Iran |
| **Web of Science** | 1- “Depression “OR “Depressive “OR “**Depress*”OR “**Anxiety”OR “Anxieties “OR “Anxiousness”OR “Nervousness”OR “Hypervigilance”OR “**Sleep**”OR “Mental Disorders”OR “Mental Health**”OR “**insomnia”  2- “Medical students”OR “Medical student”OR “resident doctors”OR “resident doctor”OR “resident physicians”OR “resident physician”OR “Residency”  3- Iran |
