# Supplementary material for: The prevalence of depression, anxiety, and sleep disturbances among medical students and resident physicians in Iran: A systematic review and meta-analysis
Source: PLoS One. 2024 Aug 23;19(8):e0307117. doi: 10.1371/journal.pone.0307117 (PMC11343466; doi:10.1371/journal.pone.0307117)
Supplement: S3 Table — (DOCX) [file pone.0307117.s003.docx]

**Supporting Table 3.** The characteristics of included studies from Iranian national databases.

| **Author** | **Year** | **Study design** | **Medical School** | **Population** | **Total Patients (Total/ Those who respond)** | **Age (mean ± SD)** | **Male (%)** | **Sampling method** | **Questionnaire** | **Rate of response** | **Assessment scales and cutoff value** |
| --- | --- | --- | --- | --- | --- | --- | --- | --- | --- | --- | --- |
| Bidokhti et al. (1) | 2010 | Cross-sectional | Birjand University of Medical Sciences | Medical Students (combined) | (175/151) | 22± 2 | 41% | counting | BDI-13 | 86.20% | BDI>4 |
| Abedini et al. (2) | 2008 | Cross-sectional | Hormozgan University of Medical Sciences | Medical Students (combined) | (nr/95) | NA | NA | systematic random sampling | BDI-21 | NA | BDI>9 |
| Aghakhani et al. (3) | 2016 | Cross-sectional | Urumia University of Medical Sciences | Medical Students (combined) | (90/90) | NA | 41 | convenience | University student depression inventory (USDI) | 100 | NA |
| Zahra Miri et al. (4) | 2021 | Cross-sectional | Hamadan University of Medical Sciences | Medical Students (clinical) | (nr/257) | 24.09±1.61 | 44.4 | counting | DASS-21, Item Insomnia Severity Index-7 | NA | Depression>9  Anxiety>7  Item Insomnia Severity Index-7>7 |
| Labbafi-nejad et al. (5) | 2010 | Cross-sectional | Tehran University of Medical Sciences | Medical Students (combined) | 196 | NA | 0 | stratified | Spielberger STAI (state-trait anxiety inventory) | NA | NA |
| Farhadi-nasab et al. (6) | 2007 | Cross-sectional | Hamedan University of Medical Sciences | Medical Students (combined) | 150 | 21.73 ± 3.5 range=18-32 | 52% | random | PSQI | NA | NA |
| Ardani et al. (7) | 2011 | Cross-sectional | Mashhad University of Medical Sciences | Medical Students (combined) | 310 /231 | 22.7 ± 2.6 range=18-40 | 28% | random | PSQI | 74% | PSQI>5 |
| Soltani-far et al. (8) | 2010 | Cross-sectional | Mashhad University of Medical Sciences | Medical Students (clinical) | 100 / 92 (46 intern + 46 stager) | NA | 0 | random | Spielberger anxiety test | 92% | SAT>20 |
| Darabi et al. (9) | 2021 | Cross-sectional | Islamic Azad University of Medical Sciences | Medical Students (clinical) | 140/121 | 20± 2 | 31% | counting | DASS-21, PSQI | 86% | PSQI>5; DASS-21: NR |
| Hashemi et al. (10) | 2001 | Cross-sectional | Yasuj University of Medical Sciences | Medical Students (combined) | Nr/86 | NR | NR | random | BDI | NR | 10-19: mild; 20-29: moderate; 30-39: severe; 40> very severe |
| Akbar et al. (11) | 2015 | Cross-sectional | Jahrom University of Medical Sciences | Medical Students (combined) | Nr/91 | NR | NR | convenience | BDI | NR | 14-19: mild; 20-28: moderate; 29-63: severe |

1. ناهید رحمانی ب, محمود صادقی خ, بیتا ب. مقایسه شیوع افسردگی در دانشجویان پزشکی، در سال های اول و آخر دانشگاه علوم پزشکی بیرجند. دانشگاه علوم پزشکی بیرجند. 1393;سال بيست و يكم(2):246-52.

2. سمیره ع, اقدس د, فائزه ص, دکتر محمود م, دکتر امید ص. شیوع افسردگی در دانشجویان پرستاری و پزشکی دانشگاه علوم پزشکی هرمزگان. پزشکی هرمزگان. 1386;سال يازدهم(2):7-.

3. نادر آ, مهدی ت, افشین ح, وحید علی ن, غلامرضا اسم ح, علیرضا نیکونژاد. ارتباط بین سلامت معنوی و افسردگی در دانشجویان پزشکی دانشگاه علوم پزشکی ارومیه. پزشکی ارومیه.سال بيست و هفتم:642-9.

4. Miri Z, Razavi Z, Mohammadi SJAJoCM. Evaluation of stress, anxiety, depression, and sleep disorders in medical students of Hamadan university of medical sciences, Iran, during the COVID-19 pandemic. 2021;27(4):232-8.

5. یاسر لبافی ن, آسیه بساق ز. بررسی شیوع اضطراب و عوامل موثر بر آن در دانشجویان دختر رشته پزشکی عمومی. سلامت کار ایران. 1391;سال نهم(3):32-8.

6. نسب ف, عبداالله, عظیمی, سینا حJمپبا. بررسی الگو و کیفیت ذهنی خواب در دانشجویان پزشکی دانشگاه علوم پزشکی همدان و ارتباط آن با صفات شخصیتی. 2008;15(1):11-5.

7. طلایی. بررسی نقش عوامل جمعیت شناختی و شاخص توده ی بدنی بر کیفیت خواب دانشجویان پزشکی. مجله اصول بهداشت روانی. 2012;14(54):9-132.

8. عاطفه سف, فاطمه م, ابراهيم ع, بهاره ت. بررسي نشانه هاي اضطراب در دانشجويان دختر رشته هاي پزشکي و پيراپزشکي دانشگاه علوم پزشکي مشهد.

9. رافعه مومنی د, محمد معیل ا. بررسی ارتباط کیفیت خواب و سطح استرس, اضطراب, افسردگی در میان دانشجویان پزشکی دانشکده دانشگاه آزاد اسلامی ساری. اولین کنفرانس بین المللی تحقیقات پیشرفته در مدیریت و علوم انسانی1400.

10. سیدنذیر هاشمی م, علی ک. بررسی میزان شیوع افسردگی در دانشجویان دانشگاه علوم پزشکی یاسوج. ارمغان دانش. 1380;سال ششم(1-2):14-.

11. کلانی, نوید, اکبر ا, کوتی, احمدابادی ن, مصیب, زانکو زمJمعپ. بررسی شیوع افسردگی در دانشجویان دانشگاه علوم پزشکی جهرم در سال 1392. 2015;15(47):58-66.
