# Supplementary material for: The prevalence of depression, anxiety, and sleep disturbances among medical students and resident physicians in Iran: A systematic review and meta-analysis
Source: PLoS One. 2024 Aug 23;19(8):e0307117. doi: 10.1371/journal.pone.0307117 (PMC11343466; doi:10.1371/journal.pone.0307117)
Supplement: S4 Table — (DOCX) [file pone.0307117.s004.docx]

**Supporting Table 4.** Quality assessment for each included study. Only studies with available full-texts were subjected for assessment of quality.

| **Study** | **Representativeness** | **Sample Size** | **Non-Respondents** | **Valid Measurement  Tool** | **Valid Statistical  Methods** | **Peer reviewed** | **Overall score** |
| --- | --- | --- | --- | --- | --- | --- | --- |
| **Abdali et al.** | **1** | **1** | **0** | **1** | **1** | **1** | **5** |
| **Aghajani Liasi et al** | **1** | **0** | **1** | **1** | **1** | **1** | **5** |
| **Ashouri et al.** | **1** | **0** | **1** | **1** | **1** | **1** | **5** |
| **Ghoreishi et al** | **1** | **1** | **1** | **1** | **0** | **1** | **5** |
| **Haghighi, M.** | **1** | **0** | **1** | **1** | **1** | **1** | **5** |
| **Jafari, N.** | **1** | **1** | **1** | **1** | **1** | **1** | **6** |
| **Janatmakan Amiri, A.** | **1** | **1** | **1** | **0** | **1** | **1** | **5** |
| **Javadi, A. H. S.** | **1** | **1** | **1** | **1** | **1** | **1** | **6** |
| **Khorvash, F.** | **1** | **1** | **1** | **0** | **1** | **1** | **5** |
| **Maghsoudi, S.** | **1** | **0** | **1** | **1** | **1** | **1** | **5** |
| **Malek et al.** | **1** | **0** | **1** | **0** | **1** | **1** | **4** |
| **Miri et al** | **1** | **1** | **1** | **1** | **1** | **1** | **6** |
| **Mohammadbeigi, A.** | **1** | **1** | **1** | **1** | **1** | **1** | **6** |
| **Moudi et al.** | **1** | **0** | **0** | **1** | **1** | **1** | **4** |
| **Naderi et al.** | **1** | **1** | **0** | **1** | **1** | **1** | **5** |
| **Nakhostin-Ansari, A.** | **1** | **1** | **0** | **1** | **1** | **1** | **5** |
| **Pournik et al.** | **1** | **0** | **0** | **1** | **1** | **1** | **4** |
| **Rahmati, F.** | **1** | **0** | **1** | **1** | **1** | **1** | **5** |
| **Rezaei et al** | **1** | **1** | **1** | **1** | **1** | **1** | **6** |
| **Sadr, S. S.** | **1** | **0** | **1** | **1** | **1** | **1** | **5** |
| **Sahraian** | **1** | **0** | **0** | **1** | **1** | **1** | **4** |
| **Shadzi et al** | **1** | **1** | **1** | **1** | **1** | **1** | **6** |
| **Shariatpanaahi, M. V.** | **1** | **0** | **0** | **1** | **1** | **1** | **4** |
| **Teimouri et al** | **1** | **1** | **0** | **1** | **1** | **1** | **5** |
| **Yazdi, Z.** | **1** | **0** | **1** | **1** | **1** | **1** | **5** |
| **Bidokhti** | **1** | **0** | **1** | **1** | **1** | **1** | **5** |
| **Abedini** | **1** | **0** | **1** | **1** | **1** | **1** | **5** |
| **Aghakhani** | **1** | **0** | **1** | **0** | **1** | **1** | **4** |
| **Zahra Miri** | **1** | **0** | **1** | **1** | **1** | **1** | **5** |
| **Labbafi-nejad et al** | **1** | **1** | **0** | **1** | **1** | **1** | **5** |
| **Farhadi-nasab et al** | **1** | **1** | **0** | **1** | **1** | **1** | **5** |
| **Ardani et al** | **1** | **0** | **0** | **1** | **1** | **1** | **4** |
| **Soltani-far et al** | **1** | **0** | **1** | **1** | **1** | **1** | **5** |
| **Darabai et al.** | **1** | **1** | **1** | **1** | **1** | **1** | **6** |
| **Hashemi et al.** | **1** | **0** | **0** | **1** | **1** | **1** | **4** |
| **Akbar et al.** | **1** | **0** | **0** | **1** | **1** | **1** | **4** |
