# Supplementary figures and images for: The prevalence of depression, anxiety, and sleep disturbances among medical students and resident physicians in Iran: A systematic review and meta-analysis
Source: PLoS One. 2024 Aug 23;19(8):e0307117. doi: 10.1371/journal.pone.0307117 (PMC11343466; doi:10.1371/journal.pone.0307117)

**Supporting Figure 1.** Results of subgroup analysis for depression.

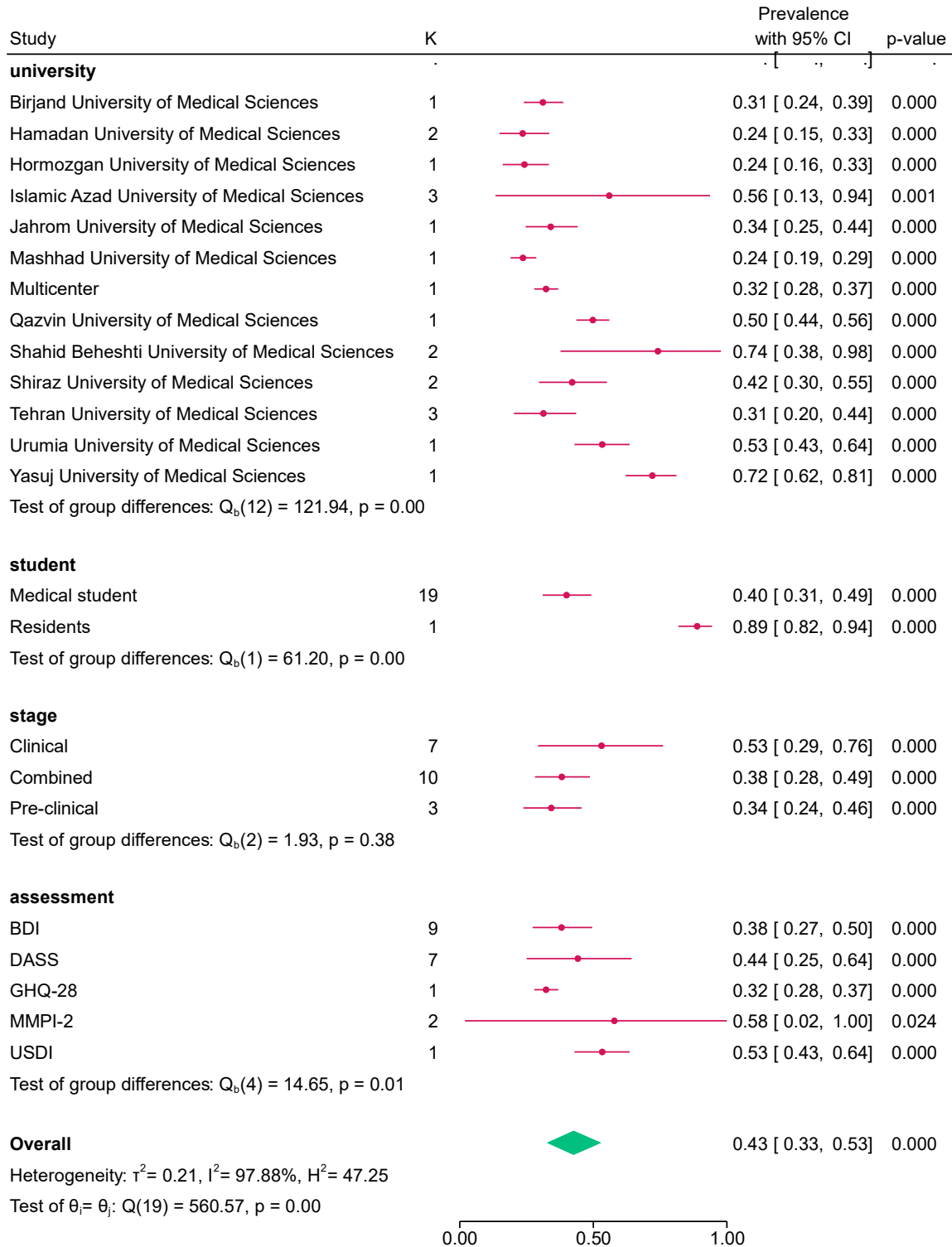

Random-effects REML model

Supplement: S1 Fig — (PDF) [file pone.0307117.s005.pdf]

**Supporting Figure 3.** Results of subgroup analysis for anxiety.

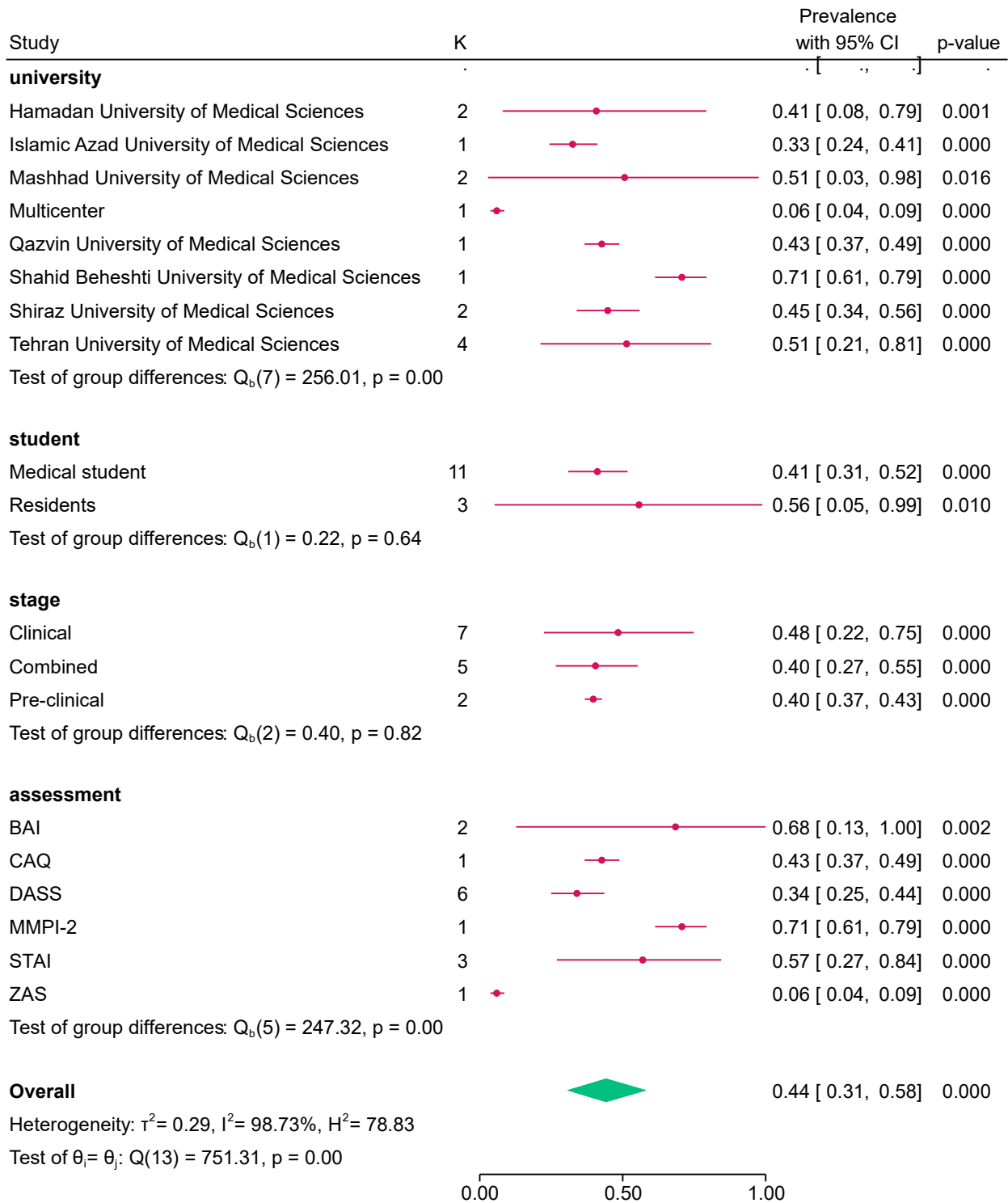

Random-effects REML model

Supplement: S3 Fig — (PDF) [file pone.0307117.s007.pdf]

**Supporting Figure 5.** Results of subgroup analysis for sleep disorder.

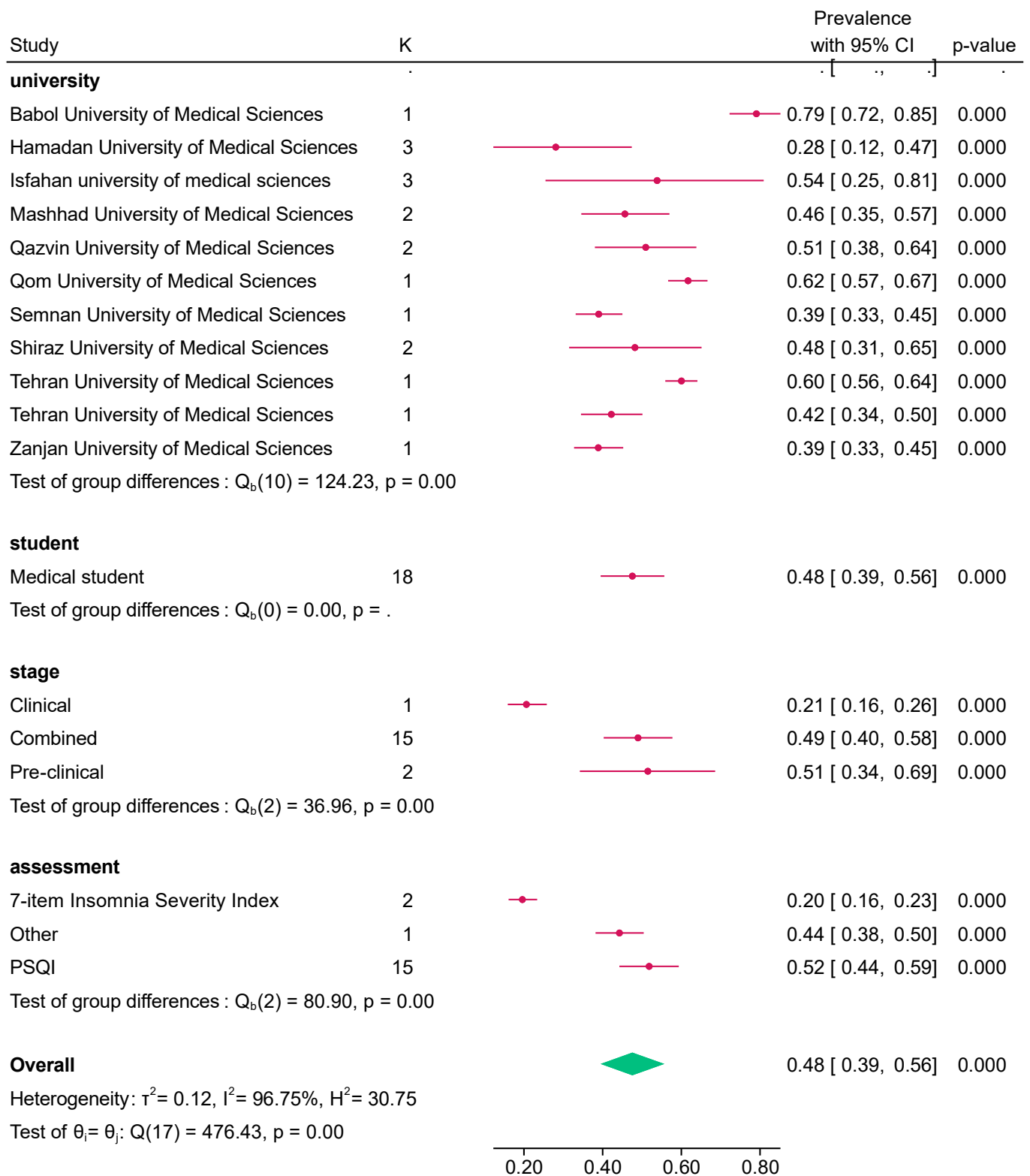

Random-effects REML model

Supplement: S5 Fig — (PDF) [file pone.0307117.s009.pdf]
