# Supplementary material for: The prevalence of depression, anxiety, and sleep disturbances among medical students and resident physicians in Iran: A systematic review and meta-analysis
Source: PLoS One. 2024 Aug 23;19(8):e0307117. doi: 10.1371/journal.pone.0307117 (PMC11343466; doi:10.1371/journal.pone.0307117)
Supplement: S2 Fig — (PDF) [file pone.0307117.s006.pdf]

Supporting Figure 2. Results of meta-analysis analysis for moderate to severe depression.

Study	Number of successes	Total	Prevalence with 95% CI	Weight (%)
Aghajani Liasi et al.	27	120	0.23 [0.15, 0.30]	8.28
Haghighi, M.	39	207	0.19 [0.14, 0.24]	8.40
Jafari et al.	117	477	0.25 [0.21, 0.28]	8.49
Nakhostin-Ansari, A.	35	323	0.11 [0.08, 0.14]	8.46
Rahmati, F.	54	99	0.55 [0.45, 0.64]	8.23
Rezaei et al	139	553	0.25 [0.22, 0.29]	8.50
Bidokhti et al.	32	151	0.21 [0.15, 0.28]	8.34
Abedini et al.	7	95	0.07 [0.03, 0.14]	8.21
Aghakhani et al.	13	90	0.14 [0.08, 0.23]	8.19
Zahra Miri et al.	34	257	0.13 [0.09, 0.18]	8.43
Darabi et al.	105	121	0.87 [0.80, 0.92]	8.29
Hashemi et al.	14	86	0.16 [0.09, 0.25]	8.18
**Overall**			0.25 [0.14, 0.38]	

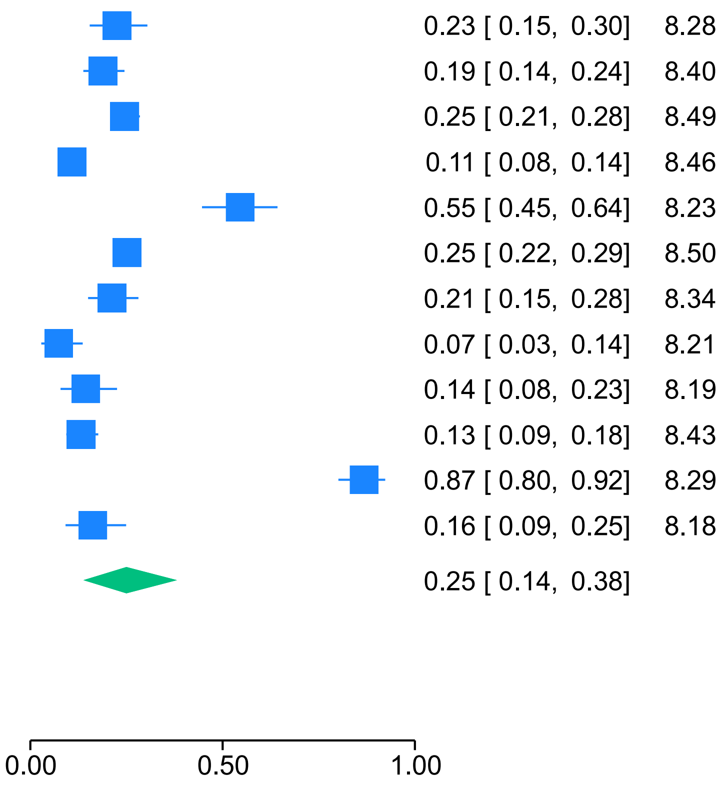

Heterogeneity: τ^2= 0.24, I^2= 98.03%, H^2= 50.83

Test of $\theta_i = \theta_j$: Q(11) = 356.63, p = 0.00

Test of θ = 0: z = 6.72, p = 0.00

Random-effects REML model
