# Supplementary material for: The prevalence of depression, anxiety, and sleep disturbances among medical students and resident physicians in Iran: A systematic review and meta-analysis
Source: PLoS One. 2024 Aug 23;19(8):e0307117. doi: 10.1371/journal.pone.0307117 (PMC11343466; doi:10.1371/journal.pone.0307117)
Supplement: S4 Fig — (PDF) [file pone.0307117.s008.pdf]

Supporting Figure 4. Results of meta-analysis analysis for moderate to severe anxiety.

Study	Number of successes	Total	Prevalence with 95% CI	Weight (%)
Aghajani Liasi et al.	25	120	0.21 [0.14, 0.29]	10.94
Ashouri et al.	86	130	0.66 [0.58, 0.74]	10.97
Haghighi, M.	182	297	0.61 [0.56, 0.67]	11.18
Jafari et al.	96	477	0.20 [0.17, 0.24]	11.24
Khorvash, F.	22	370	0.06 [0.04, 0.09]	11.21
Nakhostin-Ansari, A.	46	323	0.14 [0.11, 0.18]	11.19
Rahmati, F.	37	99	0.37 [0.28, 0.47]	10.85
Rezaei et al	160	553	0.29 [0.25, 0.33]	11.26
Zahra Miri et al.	35	257	0.14 [0.10, 0.18]	11.15
**Overall**			0.28 [0.15, 0.43]	

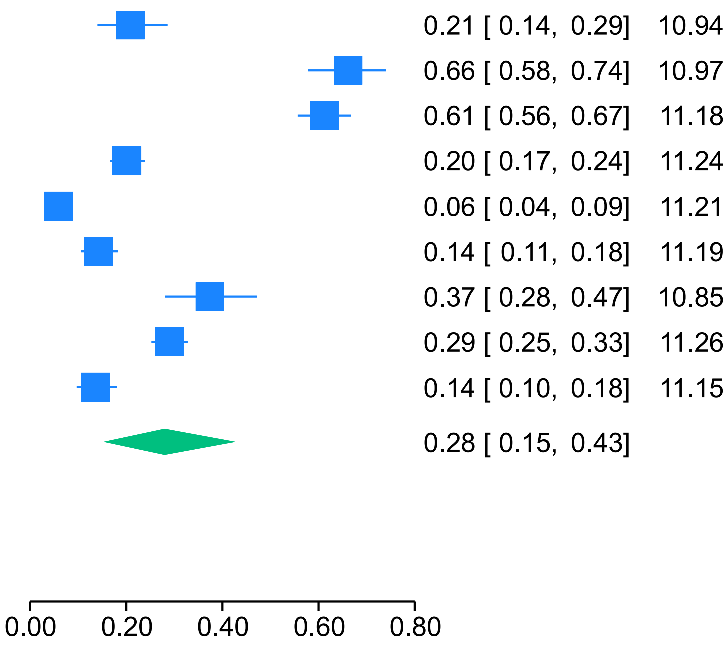

Heterogeneity: $\tau^2 = 0.22$, $I^2 = 98.42\%$, $H^2 = 63.18$

Test of $\theta_i = \theta_j$: $Q(8) = 437.50$, $p = 0.00$

Test of $\theta = 0$: $z = 6.63$, $p = 0.00$

Random-effects REML model
